# Design, synthesis and evaluation of novel 3,5-disubstituted benzamide derivatives as allosteric glucokinase activators

**DOI:** 10.1186/s13065-019-0532-8

**Published:** 2019-01-28

**Authors:** Ajmer Singh Grewal, Rajeev Kharb, Deo Nandan Prasad, Jagdeep Singh Dua, Viney Lather

**Affiliations:** 1 Chitkara College of Pharmacy, Chitkara University, Rajpura, Punjab 140401 India; 2 I. K. Gujral Punjab Technical University, Jalandhar, Punjab 144601 India; 3 Amity Institute of Pharmacy, Amity University, Noida, U.P 201303 India; 4 Shivalik College of Pharmacy, Naya-Nangal, Punjab 140126 India; 5 Jan Nayak Ch. Devi Lal Memorial College of Pharmacy, Sirsa, Haryana 125055 India

**Keywords:** Antidiabetic activity, Benzamides, Glucokinase, GK activators, In silico, In vitro assay, OGTT

## Abstract

Glucokinase (GK) is the key enzyme expressed in β-cells of pancreas and liver hepatocytes and helps in the maintenance of blood glucose levels in normal range. Activators of GK are the novel category of drug candidates which activate GK enzyme allosterically and show their antidiabetic activity. A new series of 3,5-disubstituted benzamide analogues was designed, synthesized and evaluated as GK activators by in vitro assay as well as in silico docking studies followed by evaluation of antihyperglycemic activity in animal model. Amongst the synthesized derivatives, compounds **5c**, **5f**, **5i**, **6c**, **6e** and **6h** displayed excellent in vitro GK activation. Compounds **6c** and **6e** exhibited highest antihyperglycemic activity in oral glucose tolerance test in animal model. Compound **6e** displayed most significant antihyperglycemic activity and comparable to that of standard drug in animal studies. In addition, antihyperglycemic activity of the synthesized molecules was further supported by the in silico docking studies of the synthesized derivatives in the allosteric site of GK protein.
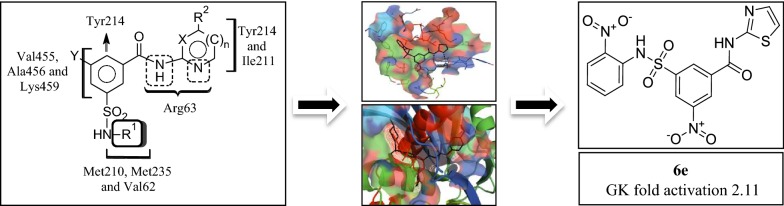

## Introduction

**Fig. 1:**
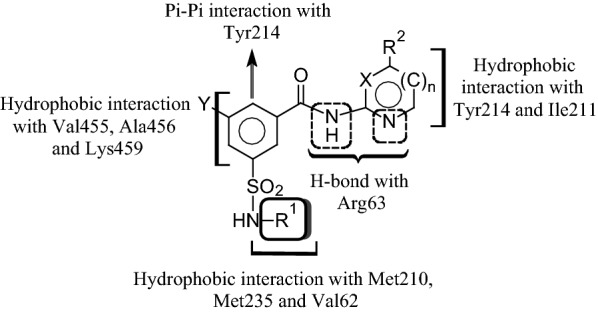
General structure of the newly designed 3,5-disubstituted benzamide GK activators and possible binding interaction with GK protein

Diabetes mellitus, often simply known as diabetes is a long-lasting disorder of food metabolism characterized by hyperglycemia due to defect in insulin secretion, insulin function or together resulting in vascular and tissue damage leading to a variety of complications of diabetes related to kidney, eyes and nerves [1–5]. Type 2 diabetes (T2D) affects more than 90% of all the diabetic patients, and is a long-term malady of food metabolism bring about due to declined insulin function [6, 7]. Despite the fact that various types of oral hypoglycaemic drugs are existing intended for the T2D management, in majority of T2D patients no single drug is helpful in achieving long-term management of blood glucose under normal physiological range. Owing to the above reason, currently physicians prescribe combination of antidiabetic drugs at an early stage for the therapeutics of T2D. Moreover, overdose of antihyperglycemic agents may lead to severe hypoglycaemia resulting in brutal toxic and side effects, and patients usually need urgent therapeutic treatment [7]. The medicinal chemists are currently working on discovering new potent antidiabetic medicines having pharmacologically different mechanism of action which can be used for single drug therapy of T2D with better safety profile. Results from numerous latest reports, including promising clinical information, have indicated that small-molecule allosteric glucokinase (GK) activators can be used to acquire these aims [8–10]. GK accelerates the breakdown of glucose to glucose-6-phosphate in presence of adenosine triphosphate (ATP) in cytoplasm and helps in the maintenance of the normal blood glucose levels in humans. GK enzyme is expressed primarily in β-cells of the pancreas and liver hepatocyte [9–11]. GK controls glucose-stimulated insulin secretion in β-cells of pancreas and metabolism of sugars in liver hepatocyte cells. GK is a promising target for the therapeutic management of T2D patients as it plays a major function in the regulation of carbohydrate breakdown. GK activators are the novel class of therapeutic agents which activate GK enzyme and show their hypoglycemic activity [8, 12–14]. A wide variety of small molecule derivatives including benzamide analogues [15–29], carboxamide derivatives [30–35], acrylamide derivatives [36], benzimidazole analogues [37, 38], quinazolines derivatives [39], thiazole derivatives [40], pyrimidine derivatives [41], and urea derivatives [42, 43] were developed in last decade to act as effective allosteric GK activators with potential antidiabetic effects. Several GK activators had been advanced to phase II clinical trials including Piragliatin, AZD6370, AZD1656, MK-0941, and AMG151; potent glucose lowering efficacy had been reported, and potential burdens have also been observed, including hypoglycaemia and increased triglyceride levels [11]. Various types of benzamide derivatives were reported as potent GK activators and the maximum drug discovery and development programmes linked to benzamide GK activators were primarily focused on the 3,5-disubstituted benzamide derivatives [16, 20–26, 44–51] possibly due to their orientation in the allosteric site and complementary binding pattern with the allosteric site amino acid residues of GK protein. In view of the significant contribution of the GK activators in the management of various diabetic disorders and the potential of 3,5-disubstituted benzamides as allosteric GK activators, we had made an attempt to design and synthesize some novel GK activators based on 3,5-disubstituted benzamide scaffold. The substitutions on benzamide nucleus were carried out in such a way that appreciable H-bond and hydrophobic interactions of the designed molecules with the amino acid residues in the allosteric site of GK protein can be achieved based on pharmacophoric requirements for binding of the ligands with GK protein (Fig. 1).

## Materials and methods

All the chemicals, reagents, solvents and proteins required during research work were procured from SRL Pvt. Ltd. (Thane), Spectrochem Pvt. Ltd. (Thane), Sigma-Aldrich (Bangalore), Merck Pvt. Ltd. (Mumbai), S.D. Fine-Chem Ltd. (Mumbai), LOBA Chemie (Mumbai) and Fisher Scientific (Mumbai) etc., and used as such. Veego VMP-D melting point apparatus was used for melting point determination of synthesized derivatives. Thin layer chromatography (TLC) was used for monitoring the reaction completion using silica gel-G. Shimadzu IR affinity FTIR spectrophotometer (KBr pellet technique) was used for recording IR spectra. BrukerAvance II 300 MHz NMR spectrophotometer was used for recording ^1^H Nuclear magnetic resonance (^1^H-NMR) and ^13^C-NMR spectra using DMSO-d_6_ as solvent and presented in parts per million (δ, ppm) downfield from internal standard (tetramethylsilane). Mass spectra were recorded on Waters Q-TOF Micromass spectrometer (ESI–MS).

### Synthesis of sulfamoyl benzamide derivatives

Dry 3-nitrobenzoic acid (0.01 mol) was taken in a round bottom flask fixed with a magnetic stirrer and the temperature was kept constant between 10 and 15 °C using cold water bath. Chlorosulphonic acid (8.0 mL) was introduced cautiously and checked to substantiate no leakage. After acid had been dissolved and the exothermic reaction had been over, the reaction flask was heated on water bath at 70–80 °C for 2 h to complete the reaction followed by cooling the flask. The contents of flask were added to 150 g crushed ice with stirring to break the lumps and precipitates of 3-(chlorosulphonyl)-5-nitrobenzoic acid were filtered under vacuum followed by washing with cold water and air dried. The product obtained above (0.01 mol) was refluxed with commercially available amines (0.01 mol) in chloroform until reaction completion as monitored by TLC on silica gel G followed by cooling and precipitates of respective sulphonamides were dried. The various sulphonamides (0.01 mol) were refluxed with thionyl chloride (0.01 mol) for 3 h and excess SOCl_2_ was distilled off to get the respective benzoyl chlorides. Benzoyl chloride (1 mmol) obtained above was refluxed with 2-aminopyrimidine (1.5 mmol) and 2-aminothiazole (1.5 mmol) in chloroform. The final product (benzamide derivatives) received after the evaporation of acetone was purified by recrystallization using ethyl alcohol [24, 26, 52].

#### 3-Nitro-5-(phenylsulfamoyl)-*N*-(pyrimidin-2-yl)benzamide (**5a**)

Black solid; yield—56%; Mp (°C) 140–142; ^1^H-NMR (δ ppm, DMSO-d_6_): 8.85 (s, 1H, NH, CO–NH), 8.04–8.46 (s, 3H, CH, C_2_, C_4_ and C_6_ of C_6_H_3_CO), 6.95–8.45 (m, 3H, CH, C_4_, C_5_ and C_6_ of pyrimidin-2-yl), 6.44–7.67 (m, 5H, C_2_, C_3_, C_4_, C_5_ and C_6_ of C_6_H_5_), 2.33 (s, 1H, NH, SO_2_NH); IR (KBr Pellets) ν cm^−1^: 3328.21 (NH str., CO–NH), 3257.77 (NH str., SO_2_–NH), 3012.81 (CH str., aromatic), 1670.35 (C=O str., CO–NH), 1624.06 (NH bend, Ar–NH), 1579.70 (C=N str.), 1533.48 (NO_2_ sym. str.), 1483.26 (C=C str., aromatic), 1409.96 (NO_2_ asym. str.), 1352.10 (SO_2_ asym. str., SO_2_–NH), 1141.86 (SO_2_ sym. str., SO_2_–NH), 717.52 (CH bend, aromatic).

#### 3-[(2-Chlorophenyl)sulfamoyl]-5-nitro-*N*-(pyrimidin-2-yl)benzamide (**5b**)

Reddish brown solid; yield—63%; Mp (°C) 157–158; ^1^H-NMR (δ ppm, DMSO-d_6_): 8.69 (s, 1H, NH, CO–NH), 8.21–8.60 (s, 3H, CH, C_2_, C_4_ and C_6_ of C_6_H_3_CO), 6.95–8.45 (m, 3H, CH, C_4_, C_5_ and C_6_ of pyrimidin-2-yl), 6.57–7.40 (m, 4H, C_3_, C_4_, C_5_ and C_6_ of C_6_H_4_Cl), 2.50 (s, 1H, NH, SO_2_NH); ^13^C-NMR (δ ppm, DMSO-d_6_): 165.94 (C=O), 157.57 (C), 156.61 (CH), 148.34 (C), 135.83 (C), 132.93 (CH), 128.32 (C), 127.78 (CH), 124.13 (CH), 110.31 (CH); IR (KBr pellets) ν cm^−1^: 3328.21 (NH str., CO–NH), 3257.77 (NH str., SO_2_–NH), 3012.81 (CH str., aromatic), 1708.93 (C=N str.), 1674.21 (C=O str., CO–NH), 1614.42 (NH bend, Ar–NH), 1529.55 (NO_2_ sym. str.), 1409.96 (NO_2_ asym. str.), 1350.17 (SO_2_ asym. str., SO_2_–NH), 1236.37 (SO_2_ sym. str., SO_2_–NH); MS (ESI TOF) m/z for C_17_H_12_ClN_5_O_5_S [M+H]^+^ Calcd 434.032, found 434.087.

#### 3-[(3-Chlorophenyl)sulfamoyl]-5-nitro-*N*-(pyrimidin-2-yl)benzamide (**5c**)

Reddish brown solid; yield—62%; Mp (°C) 161–162; ^1^H-NMR (δ ppm, DMSO-d_6_): 8.61 (s, 1H, NH, CO–NH), 8.12–8.45 (s, 3H, CH, C_2_, C_4_ and C_6_ of C_6_H_3_CO), 6.88–8.02 (m, 3H, CH, C_4_, C_5_ and C_6_ of pyrimidin-2-yl), 6.23–7.17 (m, 4H, C_2_, C_4_, C_5_ and C_6_ of C_6_H_4_Cl), 2.54 (s, 1H, NH, SO_2_NH); IR (KBr pellets) ν cm^−1^: 3360.00 (NH str., CO–NH), 3331.07 (NH str., SO_2_–NH), 3086.11 (CH str., aromatic), 1703.14 (C=N str.), 1674.21 (C=O str., CO–NH), 1614.42 (NH bend, Ar–NH), 1529.55 (NO_2_ sym. str.), 1448.54 (NO_2_ asym. str.), 1352.10 (SO_2_ asym. str., SO_2_–NH), 1149.57 (SO_2_ sym. str., SO_2_–NH), 785.03 (C–Cl str., aromatic), 713.66 (CH bend, aromatic).

#### 3-[(4-Chlorophenyl)sulfamoyl]-5-nitro-*N*-(pyrimidin-2-yl)benzamide (**5d**)

Light brown solid; yield—61%; Mp (°C) 165–167; ^1^H-NMR (δ ppm, DMSO-d_6_): 8.74 (s, 1H, NH, CO–NH), 8.18–8.62 (s, 3H, CH, C_2_, C_4_ and C_6_ of C_6_H_3_CO), 6.92–8.46 (m, 3H, CH, C_4_, C_5_ and C_6_ of pyrimidin-2-yl), 6.52–7.44 (m, 4H, C_2_, C_3_, C_5_ and C_6_ of C_6_H_4_Cl), 2.50 (s, 1H, NH, SO_2_NH); IR (KBr pellets) ν cm^−1^: 3363.86 (NH str., CO–NH), 3182.55 (NH str., SO_2_–NH), 3086.11 (CH str., Aromatic), 1697.36 (C = N str.), 1674.21 (C=O str., CO–NH), 1616.35 (NH bend, Ar–NH), 1531.48 (NO_2_ sym. str.), 1354.03 (NO_2_ asym. str.), 1311.59 (SO_2_ asym. str., SO_2_–NH), 1143.79 (SO_2_ sym. str., SO_2_–NH).

#### 3-Nitro-5-[(2-nitrophenyl)sulfamoyl]-*N*-(pyrimidin-2-yl)benzamide (**5e**)

Yellow solid; yield—68%; Mp (°C) 155–156; ^1^H-NMR (δ ppm, DMSO-d_6_): 8.72 (s, 1H, NH, CO–NH), 8.14–8.56 (s, 3H, CH, C_2_, C_4_ and C_6_ of C_6_H_3_CO), 6.78–8.34 (m, 3H, CH, C_4_, C_5_ and C_6_ of pyrimidin-2-yl), 6.34–7.44 (m, 4H, C_3_, C_4_, C_5_ and C_6_ of C_6_H_4_NO_2_), 2.54 (s, 1H, NH, SO_2_NH); IR (KBr pellets) ν cm^−1^: 3317.57 (NH str., CO–NH), 3157.46 (NH str., SO_2_–NH), 3091.89 (CH str., aromatic), 1674.21 (C=O str., CO–NH), 1674.21 (C=N str.), 1620.21 (NH bend, Ar–NH), 1525.69 (NO_2_ sym. str.), 1483.26 (C=C str., Aromatic), 1440–83 (NO_2_ asym. str.), 1311.59 (SO_2_ asym. str., SO_2_-NH), 1157.29 (SO_2_ sym. str., SO_2_–NH), 713.66 (CH bend, aromatic).

#### 3-Nitro-5-[(3-nitrophenyl)sulfamoyl]-*N*-(pyrimidin-2-yl)benzamide (**5f**)

Pale yellow solid; yield—65%; Mp (°C) 158–160; ^1^H-NMR (δ ppm, DMSO-d_6_): 8.68 (s, 1H, NH, CO–NH), 8.26–8.56 (s, 3H, CH, C_2_, C_4_ and C_6_ of C_6_H_3_CO), 6.82–8.42 (m, 3H, CH, C_4_, C_5_ and C_6_ of pyrimidin-2-yl), 6.72–7.42 (m, 4H, C_2_, C_4_, C_5_ and C_6_ of C_6_H_4_NO_2_), 2.53 (s, 1H, NH, SO_2_NH); ^13^C-NMR (δ ppm, DMSO-d_6_): 166.42 (C=O), 136.06 (C), 157.92 (C), 157.42 (CH), 148.62 (C), 148.12 (C), 136.18 (C), 130.86 (CH), 127.02 (CH), 124.74 (CH), 111.12 (CH), 110.39 (CH), 108.19 (CH); IR (KBr pellets) ν cm^−1^: 3358.07 (NH str., CO–NH), 3180.62 (NH str., SO_2_–NH), 3093.82 (CH str., aromatic), 1674.21 (C=O str., CO–NH), 1624.06 (NH bend, Ar–NH), 1531.48 (NO_2_ sym. str.), 1448.54 (NO_2_ asym. str.), 1352.10 (SO_2_ asym. str., SO_2_–NH), 1153.43 (SO_2_ sym. str., SO_2_–NH), 719.45 (CH bend, aromatic); MS (ESI TOF) m/z for C_17_H_12_N_6_O_7_S [M+H]^+^ Calcd 445.056, found 445.016.

#### 3-Nitro-5-[(4-nitrophenyl)sulfamoyl]-*N*-(pyrimidin-2-yl)benzamide (**5g**)

Yellow solid; yield—74%; Mp (°C) 152–154; ^1^H-NMR (δ ppm, DMSO-d_6_): 8.98 (s, 1H, NH, CO–NH), 8.26–8.68 (s, 3H, CH, C_2_, C_4_ and C_6_ of C_6_H_3_CO), 7.04–8.33 (m, 3H, CH, C_4_, C_5_ and C_6_ of pyrimidin-2-yl), 6.45–7.34 (m, 4H, C_2_, C_3_, C_5_ and C_6_ of C_6_H_4_NO_2_), 2.52 (s, 1H, NH, SO_2_NH); IR (KBr pellets) ν cm^−1^: 3471.87 (NH str., CO–NH), 3360.00 (NH str., SO_2_–NH), 3089.96 (CH str., aromatic), 1674.21 (C=O str., CO–NH), 1624.06 (NH bend, Ar–NH), 1612.49 (C=N str.), 1531.48 (NO_2_ sym. str.), 1479.40 (C=C str., aromatic), 1352.10 (NO_2_ asym. str.), 1311.59 (SO_2_ asym. str., SO_2_-NH), 1151.50 (SO_2_ sym. str., SO_2_–NH), 711.73 (CH bend, aromatic).

#### 3-(Benzylsulfamoyl)-5-nitro-*N*-(pyrimidin-2-yl)benzamide (**5h**)

Light brown solid; yield—76%; Mp (°C) 138–140; ^1^H-NMR (δ ppm, DMSO-d_6_): 8.63 (s, 1H, NH, CO–NH), 8.01–8.31 (s, 3H, CH, C_2_, C_4_ and C_6_ of C_6_H_3_CO), 6.88–8.34 (m, 3H, CH, C_4_, C_5_ and C_6_ of pyrimidin-2-yl), 6.51–7.42 (m, 5H, C_2_, C_3_, C_4_, C_5_ and C_6_ of C_6_H_5_), 4.34 (s, 2H, CH of CH_2_), 2.56 (s, 1H, NH, SO_2_NH); FTIR (KBr pellets) ν cm^−1^: 3377.36 (NH str., CO–NH), 3307.92 (NH str., SO_2_–NH), 3024.38 (CH str., aromatic), 2926.01 (CH str., alkyl), 1712.43 (C=O str., CO–NH), 1624.06 (NH bend, Ar–NH), 1523.71 (NO_2_ sym. str.), 1471.69 (NO_2_ asym. str.), 1350.17 (SO_2_ asym. str., SO_2_–NH), 1122.57 (SO_2_ sym. str., SO_2_–NH), 709.80 (CH bend, aromatic).

#### 3-(Ethylsulfamoyl)-5-nitro-*N*-(pyrimidin-2-yl)benzamide (**5i**)

White solid; yield—69%; Mp (°C) 147–148; ^1^H-NMR (δ ppm, DMSO-d_6_): 8.68 (s, 1H, NH, CO–NH), 8.22–8.44 (s, 3H, CH, C_2_, C_4_ and C_6_ of C_6_H_3_CO), 6.51–7.82 (m, 3H, CH, C_4_, C_5_ and C_6_ of pyrimidin-2-yl), 7.62 (s, 1H, NH, SO_2_NH), 2.48 (s, 3H, CH, CH_3_), 1.12 (t, 3H, CH of CH_3_); ^13^C-NMR (δ ppm, DMSO-d_6_): 169.92 (C=O), 165.10 (C), 164.95 (CH), 148.12 (C), 136.76 (C), 135.32 (C), 126.92 (CH), 124.72 (CH), 108.12 (CH), 40.01 (CH_2_); IR (KBr pellets) ν cm^−1^: 3381.21 (NH str., CO–NH), 3311.78 (NH str., SO_2_–NH), 3086.11 (CH str., Aromatic), 2924.09 (CH str., alkyl), 1660.71 (C=O str., CO–NH), 1606.70 (NH bend, Ar–NH), 1529.55 (NO_2_ sym. str.), 1400 (C=C str., aromatic), 1477.47 (NO_2_ asym. str.), 1352.10 (SO_2_ asym. str., SO_2_–NH), 1141.86 (SO_2_ sym. str., SO_2_–NH), 713.66 (CH bend, aromatic); MS (ESI TOF) m/z for C_13_H_13_N_5_O_5_S [M+H]^+^ Calcd 352.071, found 353.024.

#### 3-Nitro-5-(phenylsulfamoyl)-*N*-(1,3-thiazol-2-yl)benzamide (**6a**)

Dark brown solid; yield—76%; Mp (°C) 154–160; ^1^H-NMR (δ ppm, DMSO-d_6_): 8.68 (s, 1H, NH, CO–NH), 8.24–8.48 (s, 3H, CH, C_2_, C_4_ and C_6_ of C_6_H_3_CO), 7.56 (d, 1H, CH, C_5_ of thiazol-2-yl), 7.34 (d, 1H, CH, C_4_ of thiazol-2-yl), 6.68–7.34 (m, 5H, CH of C_6_H_5_), 2.54 (s, 1H, NH, SO_2_NH); IR (KBr pellets) ν cm^−1^: 3371.57 (NH str., CO–NH), 3311.78 (NH str., SO_2_–NH), 3089.96 (CH str., Aromatic), 1724.43 (C=O str., CO–NH), 1624.06 (NH bend, Ar–NH), 1531.48 (NO_2_ sym. str.), 1392.61 (NO_2_ asym. str.), 1350.17 (SO_2_ asym. str., SO_2_-NH), 1143.79 (SO_2_ sym. str., SO_2_–NH), 715.81 (CH bend, aromatic), 653.87 (C–S str., aromatic).

#### 3-[(2-Chlorophenyl)sulfamoyl]-5-nitro-*N*-(1,3-thiazol-2-yl)benzamide (**6b**)

White solid; yield—66%; Mp (°C) 181–182; ^1^H-NMR (δ ppm, DMSO-d_6_): 8.72 (s, 1H, NH, CO–NH), 8.18–8.45 (s, 3H, CH, C_2_, C_4_ and C_6_ of C_6_H_3_CO), 6.91–8.32 (m, 3H, CH, C_4_, C_5_ and C_6_ of pyrimidin-2-yl), 6.35–7.24 (m, 4H, C_3_, C_4_, C_5_ and C_6_ of C_6_H_4_Cl), 2.52 (s, 1H, NH, SO_2_NH); IR (KBr pellets) ν cm^−1^: 3371.57 (NH str., CO–NH), 3311.78 (NH str., SO_2_–NH), 3089.96 (CH str., aromatic), 1720.50 (C=O str., CO–NH), 1701.22 (C=N str.), 1631.78 (NH bend, Ar–NH), 1523.76 (NO_2_ sym. str.), 1473.62 (NO_2_ asym. str.), 1350.17 (SO_2_ asym. str., SO_2_–NH), 1143.79 (SO_2_ sym. str., SO_2_–NH), 790.81 (C–Cl str.), 713.66 (CH bend, aromatic), 655.80 (C–S str., aromatic).

#### 3-[(3-Chlorophenyl)sulfamoyl]-5-nitro-*N*-(1,3-thiazol-2-yl)benzamide (**6c**)

Greenish solid; yield—59%; Mp (°C) 189–191; ^1^H-NMR (δ ppm, DMSO-d_6_): 9.22 (s, 1H, NH, CO–NH), 8.32–8.64 (s, 3H, CH, C_2_, C_4_ and C_6_ of C_6_H_3_CO), 7.58 (d, 1H, CH, C_5_ of thiazol-2-yl), 7.38 (d, 1H, CH, C_4_ of thiazol-2-yl), 6.56–7.10 (m, 4H, CH of C_2_, C_4_, C_5_ and C_6_ of C_6_H_4_Cl), 2.51 (s, 1H, NH, SO_2_NH); ^13^C-NMR (δ ppm, DMSO-d_6_): 170.02 (C=O), 165.18 (C), 148.22 (C), 136.42 (C), 135.04 (C), 134.72 (C), 132.92 (CH), 130.12 (CH), 126.03 (CH), 125.95 (CH), 124.42 (CH), 118.72 (CH), 117.18 (CH), 109.12 (CH); IR (KBr pellets) ν cm^−1^: 3354.21 (NH str., CO–NH), 3284.77 (NH str., SO_2_–NH), 3091.89 (CH str., aromatic), 1720.50 (C=O str., CO–NH), 1631.78 (NH bend, Ar–NH), 1531.48 (NO_2_ sym. str.), 1442.75 (NO_2_ asym. str.), 1350.17 (SO_2_ asym. str., SO_2_–NH), 1143.79 (SO_2_ sym. str., SO_2_–NH), 786.96 (C–Cl str.), 717.52 (CH bend, aromatic), 651.94 (C–S str., aromatic), MS (ESI TOF) m/z for C_16_H_11_ClN_4_O_5_S_2_ [M+H]^+^ Calcd 438.993, found 439.012.

#### 3-[(4-Chlorophenyl)sulfamoyl]-5-nitro-*N*-(1,3-thiazol-2-yl)benzamide (**6d**)

Dark brown solid; yield—77%; Mp (°C) 185–186; ^1^H-NMR (δ ppm, DMSO-d_6_): 8.66 (s, 1H, NH, CO–NH), 8.16–8.48 (s, 3H, CH, C_2_, C_4_ and C_6_ of C_6_H_3_CO), 6.90–8.40 (m, 3H, CH, C_4_, C_5_ and C_6_ of pyrimidin-2-yl), 6.46–7.28 (m, 4H, C_2_, C_3_, C_5_ and C_6_ of C_6_H_4_Cl), 2.46 (s, 1H, NH, SO_2_NH); IR (KBr pellets) ν cm^−1^: 3354.21 (NH str., CO–NH), 3284.77 (NH str., SO_2_–NH), 3091.89 (CH str., aromatic), 1726.29 (C=O str., CO–NH), 1658.78 (NH bend, Ar–NH), 1608.63 (C=N str.), 1529.55 (NO_2_ sym. str.), 1442.75 (NO_2_ asym. str.), 1352.10 (SO_2_ asym. str., SO_2_–NH), 1141.86 (SO_2_ sym. str., SO_2_–NH), 790.81 (C–Cl str.), 713.66 (CH bend, aromatic), 650.01 (C–S str., aromatic).

#### 3-Nitro-5-[(2-nitrophenyl)sulfamoyl]-*N*-(1,3-thiazol-2-yl)benzamide (**6e**)

Yellowish brown solid; yield—72%; Mp (°C) 180–182; ^1^H-NMR (δ ppm, DMSO-d_6_): 8.98 (s, 1H, NH, CO–NH), 8.34–8.76 (s, 3H, CH, C_2_, C_4_ and C_6_ of C_6_H_3_CO), 7.56 (d, 1H, CH, C_5_ of thiazol-2-yl), 7.28 (d, 1H, CH, C_4_ of thiazol-2-yl), 6.68–8.10 (m, 4H, CH of C_3_, C_4_, C_5_ and C_6_ of C_6_H_4_NO_2_), 2.50 (s, 1H, NH, SO_2_NH); ^13^C-NMR (δ ppm, DMSO-d_6_): 165.02 (C=O), 167.61 (C), 148.23 (C), 142.53 (C), 136.77 (C), 134.62 (C), 132.44 (CH), 128.90 (CH), 128.79 (CH), 119.42 (CH), 115.28 (CH), 107.78 (CH); IR (KBr Pellets) ν cm^−1^: 3498.87 (NH str., CO–NH), 3385.07 (NH str., SO_2_-NH), 3089.96 (CH str., aromatic), 1687.71 (C=O str., CO–NH), 1622.13 (NH bend, Ar–NH), 1608.63 (C=N str.), 1529.55 (NO_2_ sym. str.), 1438.90 (NO_2_ asym. str.), 1348.24 (SO_2_ asym. str., SO_2_–NH), 1145.72 (SO_2_ sym. str., SO_2_–NH), 713.66 (CH bend, aromatic), MS (ESI TOF) m/z for C_16_H_11_N_5_O_7_S_2_ [M+H]^+^ Calcd 450.017, found 450.283.

#### 3-Nitro-5-[(3-nitrophenyl)sulfamoyl]-*N*-(1,3-thiazol-2-yl)benzamide (**6f**)

Dark yellow solid; yield—68%; Mp (°C) 176–177; ^1^H-NMR (δ ppm, DMSO-d_6_): 8.64 (s, 1H, NH, CO–NH), 8.18–8.56 (s, 3H, CH, C_2_, C_4_ and C_6_ of C_6_H_3_CO), 6.92–8.48 (m, 3H, CH, C_4_, C_5_ and C_6_ of pyrimidin-2-yl), 6.53–7.42 (m, 4H, C_3_, C_4_, C_5_ and C_6_ of C_6_H_4_NO_2_), 2.53 (s, 1H, NH, SO_2_NH); IR (KBr pellets) ν cm^−1^: 3275.13 (NH str., CO–NH), 3091.89 (NH str., SO_2_–NH), 2962.66 (CH str., aromatic), 1718.58 (C=O str., CO–NH), 1627.92 (NH bend, Ar–NH), 1529.55 (NO_2_ sym. str.), 1444.68 (NO_2_ asym. str.), 1350.17 (SO_2_ asym. str., SO_2_–NH), 1141.86 (SO_2_ sym. str., SO_2_–NH), 719.45 (CH bend, aromatic), 661.58 (C–S str., aromatic).

#### 3-Nitro-5-[(4-nitrophenyl)sulfamoyl]-*N*-(1,3-thiazol-2-yl)benzamide (**6g**)

Reddish brown solid; yield—59%; Mp (°C) 182–183; ^1^H-NMR (δ ppm, DMSO-d_6_): 8.88 (s, 1H, NH, CO–NH), 8.54–8.78 (s, 3H, CH, C_2_, C_4_ and C_6_ of C_6_H_3_CO), 7.51 (d, 1H, CH, C_5_ of thiazol-2-yl), 7.22 (d, 1H, CH, C_4_ of thiazol-2-yl), 6.92–8.06 (m, 4H, CH of C_2_, C_3_, C_5_ and C_6_ of C_6_H_4_NO_2_), 2.58 (s, 1H, NH, SO_2_NH); IR (KBr pellets) ν cm^−1^: 3352.28 (NH str., CO–NH), 3238.48 (NH str., SO_2_–NH), 3096.75 (CH str., aromatic), 1674.21 (C=O str., CO–NH), 1631.78 (NH bend, Ar–NH), 1531.48 (NO_2_ sym. str.), 1438.90 (NO_2_ asym. str.), 1348.24 (SO_2_ asym. str., SO_2_–NH), 1143.79 (SO_2_ sym. str., SO_2_–NH), 711.73 (CH bend, aromatic), 626.87 (C–S str., aromatic).

#### 3-(Benzylsulfamoyl)-5-nitro-*N*-(1,3-thiazol-2-yl)benzamide (**6h**)

Orange solid; yield—53%; Mp (°C) 164–166; ^1^H-NMR (δ ppm, DMSO-d_6_): 9.89 (s, 1H, NH, CO–NH), 8.18–8.20 (s, 3H, CH, C_2_, C_4_ and C_6_ of C_6_H_3_CO), 7.27 (d, 1H, CH, C_5_ of thiazol-2-yl), 7.05 (d, 1H, CH, C_4_ of thiazol-2-yl), 7.05–7.26 (m, 4H, CH of C_2_, C_3_, C_4_, C_5_ and C_6_ of C_6_H_5_), 7.26 (s, 1H, NH, SO_2_NH), 4.81 (s, 2H, CH of CH_2_); ^13^C-NMR (δ ppm, DMSO-d_6_): 170.69 (C=O), 167.61 (C), 153.09 (C), 140.57 (C), 139.27 (C), 137.69 (C), 132.54 (CH), 128.95 (CH), 128.79 (CH), 115.28 (CH), 45.34 (CH_2_); IR (KBr pellets) ν cm^−1^: 3361.93 (NH str., CO–NH), 3186.40 (NH str., SO_2_-NH), 3020.53 (CH str., aromatic), 2864.29 (CH str., alkyl), 1666.50 (C=O str., CO–NH), 1614.42 (NH bend, Ar–NH), 1531.48 (NO_2_ sym. str.), 1473.48 (NO_2_ asym. str.), 1352.10 (SO_2_ asym. str., SO_2_–NH), 1118.71 (SO_2_ sym. str., SO_2_–NH), 709.80 (CH bend, aromatic), 663.51 (C-S str., aromatic), MS (ESI TOF) m/z for C_17_H_14_N_4_O_5_S_2_ [M+H]^+^ Calcd 419.047, found 420.156.

#### 3-(Ethylsulfamoyl)-5-nitro-*N*-(1,3-thiazol-2-yl)benzamide (**6i**)

Light brown solid; yield—48%; Mp (°C) 162–163; ^1^H-NMR (δ ppm, DMSO-d_6_): 8.58 (s, 1H, NH, CO–NH), 8.21–8.60 (s, 3H, CH, C_2_, C_4_ and C_6_ of C_6_H_3_CO), 6.95–8.45 (m, 3H, CH, C_4_, C_5_ and C_6_ of pyrimidin-2-yl), 7.52 (s, 1H, NH, SO_2_NH), 2.58 (s, 3H, CH, CH_3_), 1.35 (t, 3H, CH of CH_3_); IR (KBr pellets) ν cm^−1^: 3315.63 (NH str., CO–NH), 3205.69 (NH str., SO_2_-NH), 3020.53 (CH str., aromatic), 2856.58 (CH str., Alkyl), 1674.21 (C=O str., CO–NH), 1662.64 (C=N str.), 1618.28 (NH bend, Ar–NH), 1521.84 (NO_2_ sym. str.), 1483.26 (NO_2_ asym. str.), 1350.17 (SO_2_ asym. str., SO_2_–NH), 1145.72 (SO_2_ sym. str., SO_2_–NH), 715.59 (CH bend, aromatic).

### In vitro enzyme assay

The GK activity of the synthesized compounds was evaluated using a coupled reaction with glucose-6-phosphate dehydrogenase (G-6-PDH) spectrometrically [26, 53–55]. All the compounds were prepared in dimethyl sulfoxide (DMSO) and the assay was performed in a final volume of 2000 µL containing 2-(4-(2-hydroxyethyl)piperazin-1-yl)ethanesulfonic acid (25 mM, pH 7.4), glucose (10 mM), potassium chloride (25 mM), magnesium chloride (1 mM), dithiothreitol (1 mM), ATP (1 mM), NAD (1 mM), G-6-PDH (2.5 U/mL), GK (0.5 µg), and compounds under investigation (10 µM). Absorbance was measured at 340 nm after 3 min incubation period and GK activation fold by the synthesized compounds and GK fold activation was calculated compared to control (GK activation by the control (i.e., DMSO only) was considered as 100%).

### Docking studies

Docking simulations were performed in the allosteric site of GK protein with Glide 5.8 module of Schrödinger Suite 2012 extra precision mode [56–58]. The X-ray crystallographic information of GK protein with the allosteric activator was obtained from RCSB protein data bank. After studying a numbers of entries, the best entry (PDB code: 3IMX) was selected based on resolution and was used as the docking model. The 2D structures for the designed ligands were drawn in MarvinSketch and transformed to 3D with LigPrep 2.5 with OPLS2005 force field [59]. An analogous docking method was used for the molecular docking of the synthesized derivatives as described in detail in earlier publications using Glide and the ligand poses with most favorable docking score (Glide score and Glide energy) were selected [24, 25]. The binding interactions of the ligands with GK protein were analysed further for the docked poses of the ligands using PyMOL (the PyMOL molecular graphics system, Schrödinger, LLC).

### Oral glucose tolerance test (OGTT)

Healthy Sprague–Dawley rats (150–200 g) procured from Lala Lajpat Rai University of Veterinary and Animal Sciences, Hisar and kept at controlled room temperature and fed with the normal pellet diet and water ad libitum, prior to the dietary manipulation. Consent was taken from Institutional Animal Ethics Committee to conduct this study (Approval No. JCDMCOP/IAEC/07/15/30). Based on the results of in vitro GK assay and docking studies, selected synthesized derivatives (**5b**, **5f**, **5i**, **6c**, **6e** and **6h**) were evaluated in rat OGTT model. Rats were divided into different groups containing six animals in each group and all the rats were fasted overnight for at least 8 h before experiment. Control group was administered vehicle only (5% DMSO, p.o.), standard group was administered metformin (30 mg/kg, p.o.), and test groups were administered compounds **5b**, **5f**, **5i**, **6c**, **6e** and **6h** (50 mg/kg, p.o.). All the animals were loaded with glucose (3 g/kg, p.o.) 30 min after drug administration. Blood samples were collected just prior to drug administration, and 0, 30, 60, 90 and 120 min after oral glucose administration. Blood glucose level was measured immediately and glucose area under curve (AUC) was calculated from the data (from 0 to 2 h). The OGTT assay results were statistically analyzed by two-way ANOVA [26, 27, 60].

## Results and discussion

### Chemistry

**Scheme 1:**
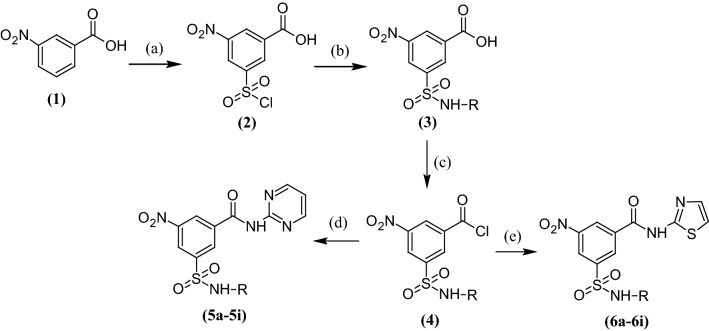
Synthetic route followed for 3,5-disubstituted benzamide derivatives. Reagents and conditions: (a) chlorosulphonic acid, 80 °C, 2 h; (b) NH_2_-R, chloroform, reflux; c thionyl chloride, chloroform, reflux; (d) 2-aminopyrimidine, chloroform, reflux; (e) 2-aminothiazole, chloroform, reflux

The synthetic pathway used for the preparation of 3,5-disubstituted benzamide derivatives is highlighted in Scheme 1. In brief, 3-(chlorosulphonyl)-5-nitrobenzoic acid (**2**) was prepared by chlorosulphonation of 3-nitrobenzoic acid (**1**) and the resultant product was reacted with amines to get the desired sulphonamide derivatives (**3**). The sulphonamides obtained above were reacted with SOCl_2_ to get respective benzoyl chlorides (**4**) which were then reacted with available heteroaromatic amines to obtain the final products (**5a**–**5i** and **6a**–**6i**). The physiochemical properties of the synthesized compounds are presented in Table 1. The synthesis as well as purity of synthesized derivatives was ensured by single spot TLC and was further established by their consistent FTIR, ^1^H and ^13^C NMR, and mass spectra.

**Table 1:** Physicochemical properties and GK activity of the synthesized 3,5-disubstituted benzamide derivatives

Compound	R	Molecular Formula	M. Pt. (°C)	R_f_^a^	% Yield	GK activity^b^
**5a**	–C_6_H_5_	C_17_H_13_N_5_O_5_S	140–142	0.62	56	1.43 ± 0.06
**5b**	2-ClC_6_H_4_–	C_17_H_12_ClN_5_O_5_S	157–158	0.37	63	1.74 ± 0.08
**5c**	3-ClC_6_H_4_–	C_17_H_12_ClN_5_O_5_S	161–162	0.53	62	1.34 ± 0.05
**5d**	4-ClC_6_H_4_–	C_17_H_12_ClN_5_O_5_S	165–167	0.57	61	1.36 ± 0.03
**5e**	2-NO_2_C_6_H_4_–	C_17_H_12_N_6_O_7_S	155–156	0.69	68	1.49 ± 0.07
**5f**	3-NO_2_C_6_H_4_–	C_17_H_12_N_6_O_7_S	158–160	0.72	65	1.89 ± 0.06
**5g**	4-NO_2_C_6_H_4_–	C_17_H_12_N_6_O_7_S	152–154	0.69	74	1.65 ± 0.04
**5h**	–CH_2_C_6_H_5_	C_18_H_15_N_5_O_5_S	138–140	0.62	76	1.20 ± 0.06
**5i**	–C_2_H_5_	C_13_H_13_N_5_O_5_S	147–148	0.56	69	1.68 ± 0.04
**6a**	–C_6_H_5_	C_16_H_12_N_4_O_5_S_2_	154–160	0.75	76	1.65 ± 0.06
**6b**	2-ClC_6_H_4_–	C_16_H_11_ClN_4_O_5_S_2_	181–182	0.56	66	1.59 ± 0.08
**6c**	3-ClC_6_H_4_–	C_16_H_11_ClN_4_O_5_S_2_	189–191	0.64	59	2.11 ± 0.05
**6d**	4-ClC_6_H_4_–	C_16_H_11_ClN_4_O_5_S_2_	185–186	0.62	77	1.55 ± 0.08
**6e**	2-NO_2_C_6_H_4_–	C_16_H_11_N_5_O_7_S_2_	180–182	0.38	72	2.11 ± 0.09
**6f**	3-NO_2_C_6_H_4_–	C_16_H_11_N_5_O_7_S_2_	176–177	0.48	68	1.37 ± 0.07
**6g**	4-NO_2_C_6_H_4_–	C_16_H_11_N_5_O_7_S_2_	182–183	0.42	59	1.24 ± 0.08
**6h**	–CH_2_C_6_H_5_	C_17_H_14_N_4_O_5_S_2_	164–166	0.55	53	1.99 ± 0.07
**6i**	–C_2_H_5_	C_12_H_12_N_4_O_5_S_2_	162–163	0.58	48	1.08 ± 0.09

^a^TLC mobile phase: Benzene: Ethyl acetate (7:3)

^b^All the values are mean of three measurements ± SD (as GK fold activation at 10 µM concentration compared to control i.e., DMSO only)

The ^1^H-NMR spectra of the synthesized benzamide derivatives showed the singlet signal equivalent to one proton of CONH functional group at around δ 9–10 ppm confirming the formation of amide linkage and presence of singlet signal for one NH proton of SO_2_NH functional group at around δ 2.5 ppm confirmed the formation of sulphonamides in the synthesized benzamide derivatives. The presence of three singlet signals at around δ 8 ppm belonging to the protons at C_2_, C_4_ and C_6_ of the phenyl ring derived from 3-nitrobenzoic acid (*meta*-nitro benzoic acid) confirmed that the amide bond, sulphonamide linkage and NO_2_ group were placed *meta* to each other i.e., separated by C_2_, C_4_ and C_6_. Due to this reason, the protons of C_2_, C_4_ and C_6_ of the phenyl ring had shown singlet signals at such a high chemical shift value. In the ^1^H-NMR spectra of compounds **5a**–**5i**, two doublet signals and one triplet signal corresponding to three aromatic CH protons were observed at around δ 8 ppm confirming the presence of pyrimidin-2-yl ring in the structure of these benzamide derivatives. In the ^1^H-NMR spectra of compounds **6a**–**6i**, two doublet signals corresponding to two aromatic CH protons were observed at around δ 7–8 ppm which confirmed that 2-amino-4-thiazol was reacted with nitro benzoyl chloride derivatives for the synthesis of respective benzamide derivatives. In the ^1^H-NMR spectra of compound **5i**, a multiplet signal for the alkyl protons in the range δ 2.5–4.0 ppm corresponding to methylene group (–CH_2_–) and a doublet signal in the range δ 1.8–2.5 ppm corresponding to protons of the methyl group confirmed the presence of ethyl group attached to SO_2_NH– in the structure of these compounds. In ^1^H-NMR spectrum of compound **35**, a doublet signal for the alkyl protons around δ 4.8 ppm and a multiplet signal around δ 6–8 ppm corresponding to five aromatic CH protons of the phenyl ring confirmed the presence of benzyl ring attached to SO_2_NH– in the structure of these compounds. In the ^1^H-NMR spectra of compound **6a**, two doublet signals and three triplet signals equivalent to five aromatic CH protons were observed at around δ 7 ppm which depicted that unsubstituted phenyl ring attached to SO_2_NH was present in the structure of these compounds. In the ^1^H-NMR spectra of compound **5b** two doublet signals and two triplet signals equivalent to four aromatic CH protons were observed in the range δ 7.1–7.6 ppm depicting the presence of 2-chlorophenyl ring attached to SO_2_NH in the structure of these compounds. In the ^1^H-NMR spectrum of compound **6c** one singlet signal, two doublet signals and one triplet signal equivalent to four aromatic CH protons were observed in the range δ 6.8–7.3 ppm indicating the presence of 3-chlorophenyl ring attached to SO_2_NH. In the ^1^H-NMR spectrum of compound **6e** two doublet signals and two triplet signals equivalent to four aromatic CH protons were observed in the range δ 6.6–8.1 ppm depicting the presence of 2-nitrophenyl ring attached to SO_2_NH. In the ^1^H-NMR spectrum of compound **5f** one singlet signal, two doublet signals and one triplet signal equivalent to four aromatic CH protons were observed indicating the presence of 3-nitrophenyl ring attached to SO_2_NH. In the ^1^H-NMR spectrum of compound **6g**, four doublet signals corresponding to four aromatic CH protons were observed in the range δ 6.9–8.1 ppm indicating the presence of 4-nitrophenyl ring attached to SO_2_NH. In the ^13^C-NMR spectra of synthesized compounds, singlet signal equivalent to carbonyl (C=O) carbon was observed at around δ 165–170 ppm indicating the presence of amide linkage (i.e., CO–NH) in the structure of the synthesized benzamide derivatives. In the ^13^C-NMR spectra of synthesized compounds, singlet signal for carbon at around δ 152 ppm confirming the presence of nitro group in the structure of synthesized compounds. In the ^13^C-NMR spectra of compounds **5b**, **5f** and **5i**; three singlet signals at around δ 157 ppm corresponding to C_2_, C_5_ and C_6_ of pyrimidine ring and one singlet signal at around δ 120 ppm were observed indicating the presence of pyrimidin-2-yl ring attached to CONH group in the structure of these compounds. A singlet signal at around δ 165 ppm corresponding to C_2_, δ 132 ppm corresponding to C_4_ and δ 109 ppm corresponding to C_5_ in the ^13^C-NMR spectra of compounds **6c**, **6e** and **6h** depicted the presence of thiazol-2-yl ring attached to CONH group in the structure of these benzamide derivatives. In the ^13^C-NMR spectra of compound **5i** singlet signals corresponding to CH_2_ and CH_3_ at around δ 40 ppm and δ 14 ppm indicated the presence of ethyl group attached to SO_2_NH group in the structure of compound **5i**. In the ^13^C-NMR spectrum of compound **6h**, a singlet signal corresponding to CH_2_ at around δ 45 ppm was observed indicating the presence methylene group (–CH_2_–) attached to SO_2_NH. The IR spectra of the synthesized benzamide derivatives showed the presence of amide NH-stretching vibrations at around 3300–3200 cm^−1^; aromatic CH-stretching vibrations above 3000 cm^−1^; SO_2_ asymmetric and symmetric stretching vibrations at around 1400–1300 cm^−1^ and 1200–1100 cm^−1^ respectively; and sulphonamide NH-stretching vibrations in the range 3400–3100 cm^−1^, thus supporting the fact that an amide linkage (CO–NH) and a sulphonamide functional group (SO_2_–NH) were present in the structure of the synthesized molecules. In the IR spectra of the synthesized molecules, C=O stretching vibrations in the range 1700–1600 cm^−1^ indicated the presence of amide carbonyl functional group in the structure of the synthesized benzamide derivatives. The NH-bending vibrations at around 1600 cm^−1^ were present in the IR spectra of the synthesized molecules confirming the presence of aromatic NH-functional group in the structure of the synthesized molecules. In the IR spectra of the synthesized molecules, CH-bending vibrations at around 800–700 cm^−1^ confirmed the presence of aromatic ring in the structure of molecules. The IR spectra of the synthesized benzamide derivatives showed the presence of NO_2_ symmetric and asymmetric stretching vibrations around 1600–1500 cm^−1^ and 1400–1300 cm^−1^ respectively supporting the presence of nitro functional group in the structure of all the synthesized molecules. In the IR spectra of compounds **5a**–**5i**, presence of C=N stretching vibrations at around 1700–1600 cm^−1^ depicted the occurrence of pyrimidine ring in these compounds. The IR spectra of compounds, **5h**, **5i**, **6h** and **6i** showed aliphatic CH-stretching vibrations in the range 3000–2800 cm^−1^ confirming the presence of aliphatic group (e.g., ethyl or benzyl) in the structure of these compounds. The IR spectra of compounds 28–36 showed the asymmetric stretching vibrations at around 1650–1600 cm^−1^ and stretching vibrations around 700–600 cm^−1^ corresponding to aromatic C–S depicting the presence of thiazole ring in the structure of these compounds.

### In vitro enzyme activity

The results of the in vitro GK assay (fold activation compared to control i.e., DMSO only) are presented in Table 1. Among the synthesized derivatives tested in vitro, compounds **5b**, **5f**, **5i**, **6c**, **6e** and **6h** showed maximum fold activation (in the range 1.7–2.1 compared to control) of GK enzyme. Compounds **5e**, **5g**, **6a**, **6b** and **6d** showed moderate fold activation (around than 1.5 compared to control) of GK enzyme. Compounds **5a**, **5c**, **5d**, **5h**, **6f** and **6g** demonstrated lower fold activation (around 1.25) of GK enzyme compared to that of control. Compound **6i** was found to be inactive in the in vitro GK assay. The results of in vitro GK assay depicted that introduction of the thiazol-2-yl ring attached to CONH resulted in increased GK activity (most potent) compared to compounds bearing pyrimidine-2-yl ring (compounds **6c** and **6e**). Amongst the compounds bearing pyrimidin-2-yl ring, the derivative bearing *N*-3-nitrophenyl sulphonamide group (compound **5f**) demonstrated highest GK fold activation of 1.89. The *N*-thiazol-2-yl substituted benzamide derivatives bearing *N*-3-chlorophenyl and *N*-2-nitrophenyl substituted sulphonamide group (compounds **6c** and **6e**) displayed highest GK fold activation of 2.11 compared to control. The results of in vitro GK assay demonstrated that replacement of the aromatic ring attached to sulphonamide NH with alkyl group such as ethyl (compound **6i**) led to reduced GK activity compared to the compounds bearing substituted aromatic ring attached to sulphonamide NH.

### Molecular docking studies

Lead optimization of the synthesized derivatives was done via calculation of drug-likeness properties (log P, mol. wt., hydrogen bond acceptors (HBA), and hydrogen bond donors (HBD) and all the synthesized derivatives showed appreciable drug-like properties as ascertained using Lipinski’s rule of 5 (Table 2). The docking studies were performed using Glide in the allosteric site of GK protein (PDB entry: 3IMX) and validated by docking of 3IMX ligand in the allosteric site. The designed GK activators were docked in the allosteric binding site comprising of Arg63, Tyr215, Met210, Tyr214, Val452 and Val455 residues. Glide score and Glide energy of the synthesized derivatives are presented in Table 2. All the synthesized 3,5-disubstituted benzamide derivatives showed significant binding in the allosteric site as determined by analysing the H-bond and hydrophobic interactions of the selected best docked poses. Based on their most favourable Glide score, lowest Glide energy (kcal/mol) and binding interactions with allosteric site residues of GK protein, compounds **5b**, **5f**, **5i**, **6c**, **6e** and **6h** were further analyzed in details using PyMOL to explore the binding mode and docking interactions of the designed molecules with the amino acid residues in the allosteric site of GK protein.

**Table 2:** Molecular properties, docking score and Glide energy of the synthesized 3,5-disubstituted benzamide molecules

Compound	M. Wt.^a^	log P^a^	HBA^a^	HBD^a^	Glide score	Glide energy
**5a**	399.38	2.25	7	2	− 9.59	− 45.95
**5b**	433.82	2.85	7	2	− 11.26	− 53.63
**5c**	433.82	2.85	7	2	− 11.23	− 50.77
**5d**	433.82	2.85	7	2	− 9.97	− 43.86
**5e**	444.38	2.19	9	2	− 10.20	− 51.40
**5f**	444.38	2.19	9	2	− 10.37	− 57.62
**5g**	444.38	2.19	9	2	− 9.91	− 56.42
**5h**	413.41	2.31	7	2	− 10.69	− 56.89
**5i**	351.34	0.95	7	2	− 10.12	− 50.57
**6a**	404.42	2.84	6	2	− 11.22	− 48.59
**6b**	438.86	3.44	6	2	− 9.96	− 53.62
**6c**	438.86	3.44	6	2	− 10.98	− 53.01
**6d**	438.86	3.44	6	2	− 10.86	− 38.77
**6e**	449.41	2.78	8	2	− 10.35	− 56.93
**6f**	449.41	2.78	8	2	− 9.86	− 56.19
**6g**	449.41	2.78	8	2	− 10.31	− 56.48
**6h**	418.44	2.97	6	2	− 11.11	− 57.19
**6i**	356.37	1.54	6	2	− 9.62	− 47.28

^a^Mol. Wt., LogP, HBA, and HBD were calculated using MarvinSketch (Marvin 15.9.21, 2015, ChemAxon, Budapest, Hungary)

**Fig. 2:**
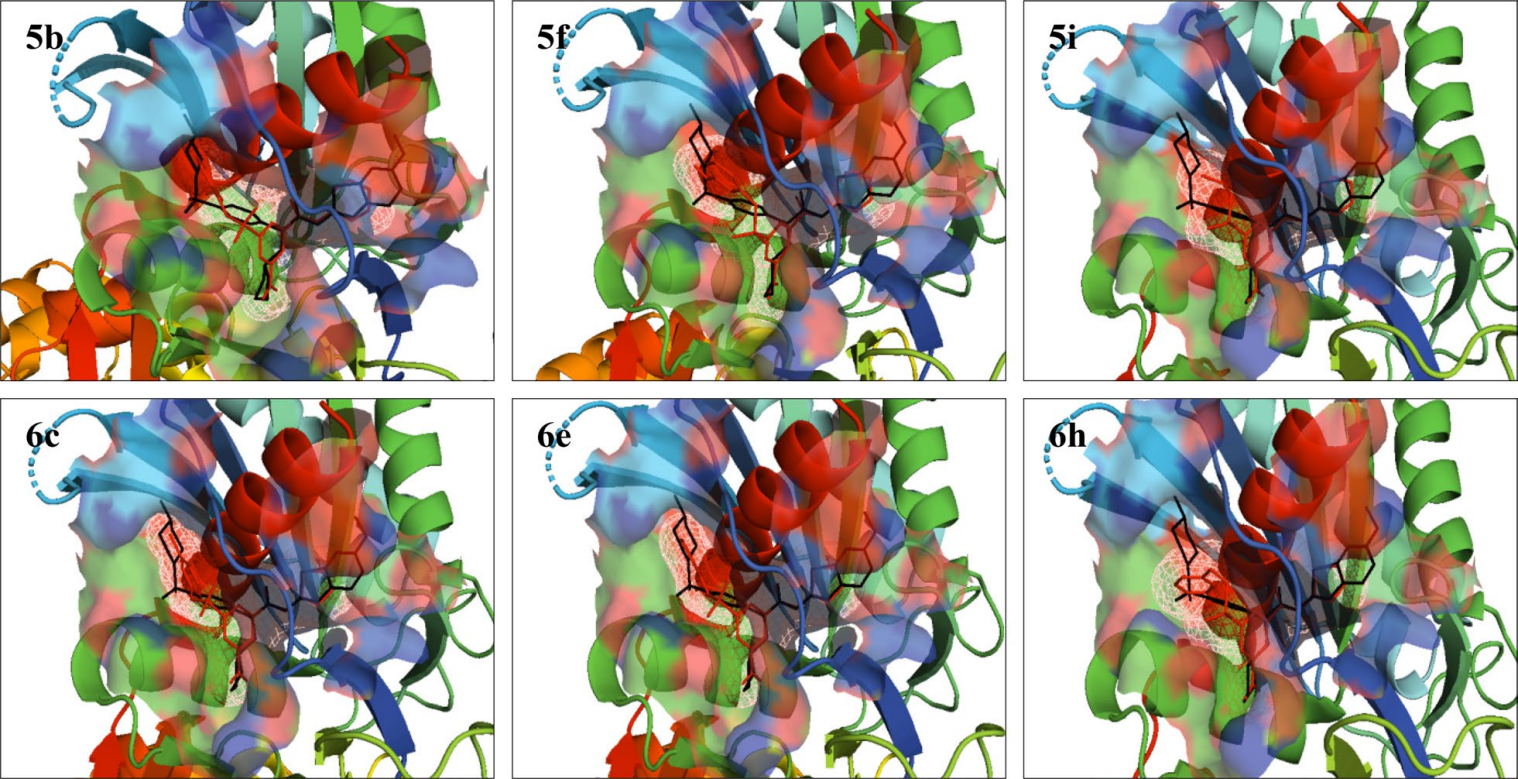
Superimpose of the docked poses for compounds **5b**, **5f**, **5i**, **6c**, **6e** and **6h** (red sticks) with that of co-crystallized ligand (PDB ID: 3IMX, black sticks) in the allosteric site of GK protein

An overlay of docked poses of the selected compounds **5b**, **5f**, **5i**, **6c**, **6e** and **6h** with that of 3IMX ligand showed that these compounds had the similar orientation and binding pattern in the allosteric binding site of GK enzyme as that of co-crystallized ligand (Fig. 2). All the selected molecules were found to bind to an allosteric pocket of GK protein, which is about 20 Å remote from the glucose binding site [61]. The pyrimidin-2-yl group attached to benzamide nucleus of compounds **5b**, **5f** and **5i** protruded in the hydrophobic pocket showing the interactions with Val455, Ala456, and Lys459 of the R13 helix, as well as Pro66 of connecting region I and Ile159 of the large domain, phenyl ring packs between Tyr214, Met210 and Val455 whereas the substituted aryl group of sulphonamide oriented into the hydrophobic pocket comprising residues Leu451, Val 455, Tyr215, and Trp99. The thiazol-2-yl group attached to benzamide nucleus of compounds **6c**, **6e** and **6h** protruded in the hydrophobic pocket showing the interactions with Val455, Ala456, and Lys459 of the R13 helix, as well as Pro66 of connecting region I and Ile159 of the large domain, the phenyl ring was found to pack between Tyr214, Met210 and Val455 whereas the substituted aryl group of sulphonamide oriented into the hydrophobic pocket comprising residues Leu451, Val 455, Tyr215, and Trp99.

**Fig. 3:**
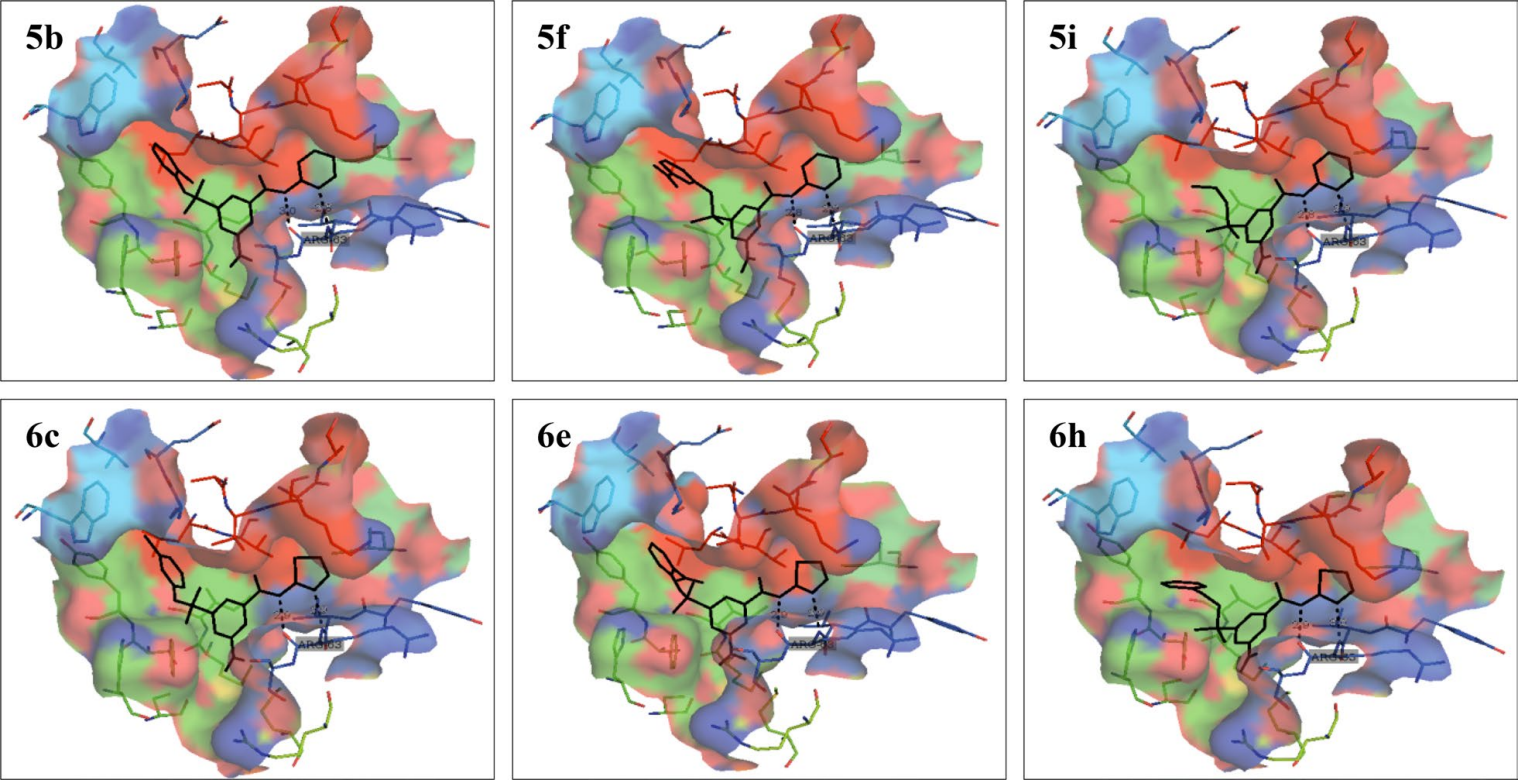
Docked poses showing H-bond interactions for compound **5b**, **5f**, **5i**, **6c**, **6e** and **6h** in the allosteric binding site of GK protein

Docked pose showing H-bond interactions of the selected compounds **5b**, **5f**, **5i**, **6c**, **6e** and **6h** in the allosteric binding site of GK protein is presented in Fig. 3. The selected 3,5-disubstituted benzamide displayed similar H-bonding interactions with amino acid residues in the allosteric binding site of GK protein as that of the co-crystallized ligand (PDB ID: 3IMX). The docked pose of compound **5b** showed the H-bond interaction between the ‘1-N’ of pyrimidin-2-yl and benzamide NH with NH and backbone carbonyl of Arg63 residue on GK protein with H-bond distance of 2.8 Å and 3.0 Å respectively. Compound **5f** showed the H-bond interaction between the ‘1-N’ of pyrimidin-2-yl and benzamide NH with NH and backbone carbonyl of Arg63 residue on GK protein with H-bond distance of 2.9 Å and 2.8 Å respectively. Compound **5i** showed the H-bond interaction between the ‘1-N’ of pyrimidin-2-yl and benzamide NH with NH and backbone carbonyl of Arg63 residue on GK protein with H-bond distance of 2.9 Å and 2.8 Å respectively. Compound **6c** showed the H-bond interaction between the ‘3-N’ of thiazol-2-yl and benzamide NH with NH and backbone carbonyl of Arg63 residue on GK protein with H-bond distance of 2.9 Å and 2.9 Å respectively. Compound **6e** showed the H-bond interaction between the ‘3-N’ of thiazol-2-yl and benzamide NH with NH and backbone carbonyl of Arg63 residue on GK protein with H-bond distance of 2.9 Å and 2.9 Å respectively. Compound **6h** showed the H-bond interaction between the ‘3-N’ of thiazol-2-yl and benzamide NH with NH and backbone carbonyl of Arg63 residue on GK protein with H-bond distance of 3.0 Å and 2.9 Å respectively. The molecular docking of the designed 3,5-disubstituted benzamide derivatives in the allosteric site of GK protein helped in understanding the mechanism of GK activation by these newly designed molecules and also helped in predicting that the designed 3,5-disubstituted benzamide derivatives could act as potent allosteric activators of GK enzyme.

### In vivo antihyperglycemic activity

**Fig. 4:**
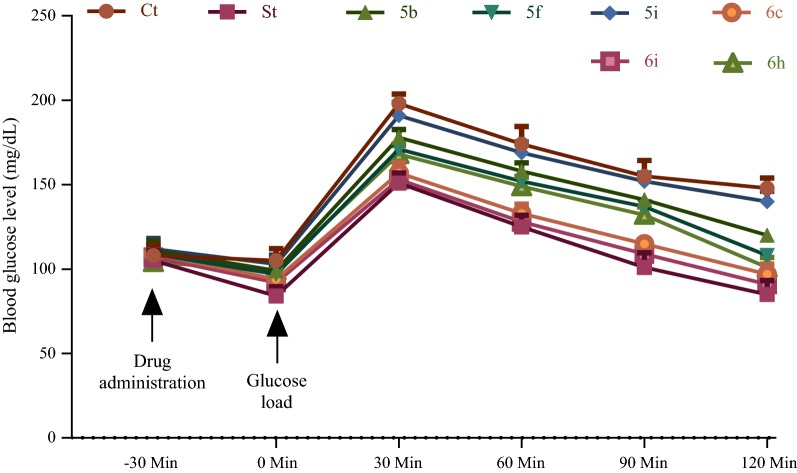
Effect of selected compounds (**5b**, **5f**, **5i**, **6c**, **6e** and **6h**) on blood glucose levels at specified time intervals in OGTT. Ct = control and St = standard. All the values are mean of six measurements ± SD. The antihyperglycemic activity data of metformin treated group and test groups were significantly different from the control group (p < 0.05); and data of all the groups was also significantly different in various time intervals compared to 0 min interval (p < 0.05)

**Fig. 5:**
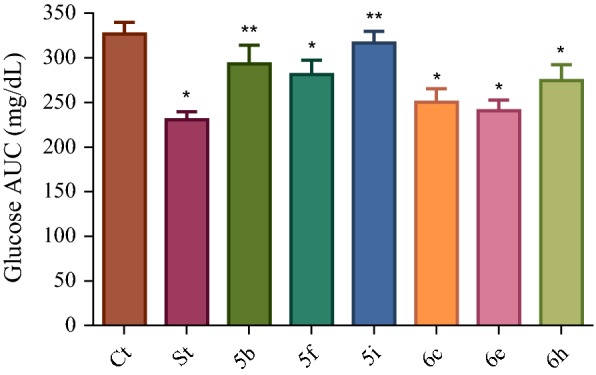
Glucose AUC reduction exhibited by the selected compounds (**5b**, **5f**, **5i**, **6c**, **6e** and **6h**) in rat OGTT model. Ct = control and St = standard. All the values are mean of six measurements ± SD. *Data were significantly different from that of control group (p < 0.05), **Data were not significantly different from that of control group as analyzed statistically by one-way ANOVA

Based on screening by in vitro GK assay and in silico docking studies selected compounds (**5b**, **5f**, **5i**, **6c**, **6e** and **6h**) were further evaluated for their glucose lowering effect in animal models by means of OGTT assay. The results of antihyperglycemic activity (i.e., OGTT assay) were measured as blood glucose levels (mg/dL) at different time intervals (0, 30, 60, 90 and 120 min after oral glucose administration) and glucose AUC represented in Figs. 4 and 5, respectively. The results of antihyperglycemic activity assay depicted that amongst compounds evaluated in OGTT assay, compounds **6c** and **6e** were found to be highly active with compound **6e** showing better potency than compounds **6c** in OGTT assay. Compound **6e** was almost equipotent to standard antidiabetic drug metformin at 30 and 60 min and decreased the blood glucose levels equivalent to that of standard antidiabetic drug metformin at 120 min interval. Compound **6e** was found to reduce significantly glucose AUC compared to control and analogous to that of standard antidiabetic drug. Compounds **5f** and **6h** displayed appreciable reduction in blood glucose levels compared to that of standard drug (metformin) in OGTT assay and these compounds significantly reduced glucose AUC compared to control group. The results of in vivo antihyperglycemic activity assay indicated that the compounds **6c**, **6e** and **6h** followed the similar pattern in blood glucose lowering as that of the standard antidiabetic drug metformin. Compound **5b** was found to be fairly effective in the in vivo antihyperglycemic activity assay compared to standard antidiabetic drug metformin at 120 min interval. Compound **5i** was least effective in reducing blood glucose level in OGTT assay compared to standard drug (St) and reduction in glucose AUC compared to control group (Ct) was not significant. All the compounds tested for antihyperglycemic activity reduced blood glucose in safe range at time interval of 120 min during OGTT assay (i.e., no hypoglycaemic effect was observed during assay period). The control group (vehicle i.e., DMSO only) didn’t produce any significant effect on blood glucose levels as well as blood glucose AUC in the OGTT assay.

The results of antihyperglycemic activity assay indicated that substitution with electron withdrawing groups like chloro and nitro at phenyl ring attached to sulphonamide led to increased antidiabetic activity which can be seen from the OGTT assay results of compounds **6c** and **6e**. These antihyperglycemic activity results were in accordance with the results obtained in case of acrylamide derivatives for reduction of blood glucose levels reported by Sidduri et al. [36]. Further the results of antihyperglycemic activity assay indicated that substitution of substituted phenyl ring at sulphonamide nitrogen with alkyl groups like ethyl led to decreased antidiabetic activity which can be seen in case of compound **5i**. Substitution of ethyl group at sulphonamide NH with benzyl group resulted in increased antihyperglycemic activity. The replacement of pyrimidine-2-yl ring (6-membered ring) with thiazol-2-yl ring (5-membered ring) resulted in an increased antihyperglycemic activity which can be seen from the OGTT assay results of compounds **6c** and **6e** compared to that of compounds **5b**, **5f** and **5i**. These antihyperglycemic activity results were supported by a similar study on substituted benzamide derivatives reported as GK activators by Iino et al. [46].

## Conclusion

A novel series of 3,5-disubstituted benzamide derivatives were designed and synthesized based on the pharmacophoric features required for binding of allosteric GK activators with GK protein and previously reported 3,5-disubstituted benzamide GK. Amongst the several synthesized derivatives, compounds **5b**, **5f**, **5i**, **6c**, **6e** and **6h** displayed appreciable GK activation profile in the in vitro enzymatic assay. In the docking studies, almost all the synthesized compounds displayed appreciable binding interaction with Arg63 of allosteric site of GK protein. Amongst the compounds tested in vivo for their antihyperglycemic activity (OGTT assay), compounds **6c** and **6e** showed highest antihyperglycemic activity. The results of the in vivo antihyperglycemic activity studies were in parallel to that of the in vitro enzyme assay and in silico docking studies. The molecular properties of these newer benzamide derivatives were also found to follow the Lipinski’s rule of five for drug-like property. These newly discovered molecules can serve as the starting hits for the development of safe, potent and orally active GK activators for the treatment of T2D.
